# Retinal Boundary Segmentation in Stargardt Disease Optical Coherence Tomography Images Using Automated Deep Learning

**DOI:** 10.1167/tvst.9.11.12

**Published:** 2020-10-13

**Authors:** Jason Kugelman, David Alonso-Caneiro, Yi Chen, Sukanya Arunachalam, Di Huang, Natasha Vallis, Michael J. Collins, Fred K. Chen

**Affiliations:** 1 Queensland University of Technology (QUT), Contact Lens and Visual Optics Laboratory, Centre for Vision and Eye Research, School of Optometry and Vision Science, Queensland, Australia; 2 Centre for Ophthalmology and Visual Science (incorporating Lions Eye Institute), The University of Western Australia, Perth, Western Australia, Australia; 3 Centre for Molecular Medicine and Innovative Therapeutics, Murdoch University, Murdoch, Western Australia, Australia; 4 Centre for Neuromuscular and Neurological Disorders, The University of Western Australia and Perron Institute for Neurological and Translational Science, Nedlands, Western Australia, Australia; 5 Department of Ophthalmology, Royal Perth Hospital, Perth, Western Australia, Australia; 6 Department of Ophthalmology, Perth Children's Hospital, Nedlands, Western Australia, Australia

**Keywords:** ABCA4, image segmentation, inherited retinal diseases, trial end point, machine learning, artificial intelligence, OCT

## Abstract

**Purpose:**

To use a deep learning model to develop a fully automated method (fully semantic network and graph search [FS-GS]) of retinal segmentation for optical coherence tomography (OCT) images from patients with Stargardt disease.

**Methods:**

Eighty-seven manually segmented (ground truth) OCT volume scan sets (5171 B-scans) from 22 patients with Stargardt disease were used for training, validation and testing of a novel retinal boundary detection approach (FS-GS) that combines a fully semantic deep learning segmentation method, which generates a per-pixel class prediction map with a graph-search method to extract retinal boundary positions. The performance was evaluated using the mean absolute boundary error and the differences in two clinical metrics (retinal thickness and volume) compared with the ground truth. The performance of a separate deep learning method and two publicly available software algorithms were also evaluated against the ground truth.

**Results:**

FS-GS showed an excellent agreement with the ground truth, with a boundary mean absolute error of 0.23 and 1.12 pixels for the internal limiting membrane and the base of retinal pigment epithelium or Bruch's membrane, respectively. The mean difference in thickness and volume across the central 6 mm zone were 2.10 µm and 0.059 mm^3^. The performance of the proposed method was more accurate and consistent than the publicly available OCTExplorer and AURA tools.

**Conclusions:**

The FS-GS method delivers good performance in segmentation of OCT images of pathologic retina in Stargardt disease.

**Translational Relevance:**

Deep learning models can provide a robust method for retinal segmentation and support a high-throughput analysis pipeline for measuring retinal thickness and volume in Stargardt disease.

## Introduction

Retinal degeneration owing to inherited or age-related diseases is the most common cause of visual loss in the Western countries.^1^^,^^2^ The advent of optical coherence tomography (OCT) has provided a unique opportunity for detailed monitoring of the slow rate of retinal cell loss through measurements of retinal thicknesses in repeated volume scans over time. The accuracy of this measurement depends on the precise segmentation of the inner and outer retinal layer boundaries in large numbers of closely spaced slices from a set of OCT volume scans. Although most OCT clinical instruments can provide accurate segmentation of retinal layers in OCT images of healthy eyes, pathologic changes associated with retinal degeneration often lead to segmentation errors that require a significantly increased amount of time for manual correction.^3^^–^^5^ In addition to the impracticality of this manual approach in a busy clinical practice, interobserver variability and human error^6^ arising from grader inexperience also pose significant limitations. Therefore, there is an unmet clinical need to improve current OCT segmentation algorithms for each type of retinal pathology to allow accurate monitoring of the rate of retinal degeneration in this era of increasing therapeutic options to arrest disease progression.^7^^,^^8^

An increasing number of studies have reported semiautomated or fully automated segmentation methods with the goal of improving the accuracy, consistency and speed of segmentation in diseased retina to replace the need for manual correction. Early versions of these methods were built around standard image processing techniques and algorithms.^9^^–^^14^ More recently, machine learning and deep learning methods have been used, including support vector machines,^15^^,^^16^ random forest classifiers,^17^ patch-based classification with convolutional neural networks^18^^–^^22^ or recurrent neural networks,^20^^,^^22^ semantic segmentation with fully convolutional (encoder–decoder) networks,^22^^–^^26^ and other deep learning methods.^27^^–^^30^ Importantly, some of these methods have been applied to OCT images from patients with age-related macular degeneration,^18^^,^^20^^,^^24^^,^^27^ diabetic retinopathy,^11^^,^^25^ macular telangiectasia type 2,^29^ diabetic macular oedema,^13^^,^^23^^,^^24^ pigment epithelium detachment,^28^ glaucoma,^15^^,^^30^ multiple sclerosis ^17^^,^^26^ retinitis pigmentosa,^31^ and neurodegenerative diseases.^32^ These diseases are characterized by variable thinning of the inner retinal layers (e.g., glaucoma and multiple sclerosis), thickening or cystic changes in the nuclear layers (e.g., macular telangiectasia type 2 and diabetic retinopathy) or focal disruption of the retinal pigment epithelium (RPE, e.g., age-related macular degeneration, macular telangiectasia, and pigment epithelium detachment). However, OCT segmentation algorithms have not been investigated in Stargardt disease despite its unique lesions, including outer retinal or subretinal flecks,^33^ outer retinal atrophy with or without RPE loss, and variable loss of choroidal architecture disrupting the Bruch's membrane contour,^34^^–^^36^ which provide challenges for commercial segmentation software. Kong et al.^37^ assessed the reproducibility of OCT retinal structure parameter measurements and noted that the complex morphology of Stargardt disease made the segmentation challenging. Strauss et al.^38^ showed that monitoring the decrease in retinal volume for Stargardt disease is possible, but stressed the need to manually correct segmentation errors in more than one-third of the OCT slices. To overcome the deficiency in commercial software and the need for time-consuming manual segmentation, Velaga et al.^39^ described an “adaptive” method in which thickness measurement was calculated based on only a subset (minimum of 25 slices) of the entire OCT volume scans (49 in total) chosen by the grader to decrease the need to manually segment all OCT scans acquired. However, this approach does not address the fundamental problem of poor segmentation performance in Stargardt disease. Therefore, there is an opportunity to apply machine learning to address this clinical need. Currently, the only application of machine learning to Stargardt disease image analysis is limited to cone detection in adaptive optics scanning light ophthalmoscope split-detection images, as described by Davidson et al.^40^

In this study, we used OCT images from patients with Stargardt disease to develop and demonstrate the use of an automated machine learning–based method to segment retinal layers. We evaluated the performance of this method by calculating the error in retinal boundary position compared with the ground truth, existing retinal segmentation tools as well as a patch-based machine learning method. The differences in retina thickness and volume from the ground truth were also analyzed and compared with the repeatability of manual segmentation in OCT images with similar RPE loss.

## Methods

### Patient and Image Data

The data consists of a range of spectral domain OCT (SD-OCT) scans from patients with various stages of Stargardt disease. Approval to identify and use SD-OCT images from patients with genetically confirmed Stargardt disease for developing segmentation methods was obtained from the Human Ethics Office of Research Enterprise, The University of Western Australia (RA/4/1/8932 and RA/4/1/7916) and the Human Research Ethics Committee, Sir Charles Gairdner Hospital (2001-053). A diagnosis of Stargardt disease was made based on clinical assessment by an ophthalmologist specializing in inherited retinal diseases (FKC) supported by characteristic multimodal retinal imaging features and genetic confirmation of biallelic mutation in the *ABCA4* gene (Australian Inherited Retinal Disease Registry).

All patients underwent SD-OCT scans using the Spectralis OCT+HRA device (Heidelberg Engineering, Heidelberg, Germany). The OCT scanning protocol consisted of 61 raster lines covering an area of 30° × 25° (8.8 mm horizontally × 7.2 mm vertically) of the macula, with 119 µm separation between each line scan. The wide scan area coverage ensures no truncation or missing B-scan within the central 6 mm diameter zone. The automated real-time algorithm was used to enhance the definition of each B-scan by averaging nine OCT images. Scans were taken in high-resolution mode, unless it was determined necessary to use high-speed mode owing to poor fixation (any low-resolution scan was resized to match the resolution of the dataset). Care was taken to ensure that the scanned area was centered at the fovea even if the preferred retinal locus was eccentric. All scans were taken by a trained retinal imaging technician. For each patient, scans were acquired in both eyes, across a number of visits, over several years. However, these visits were not spaced regularly and the quantity and spacing of visits for all participants were not necessarily the same.

Total retinal thickness, defined as the axial distance^41^ from the base of the RPE or the Bruch's membrane if RPE was absent, to the internal limiting membrane (ILM), provides an indirect measure of the number of neuronal, glial, and RPE cells in the retina. The summation of this value within the central 6 mm diameter zone of the retina (macular volume) has been proposed as a useful clinical metric in tracking disease progression.^38^ Automated segmentation of these inner and outer boundaries of the retina, in all 61 B-scans of each eye were examined and adjusted manually in the HEYEX software (HEYEX-XML, Heidelberg, Germany) by a team of image graders (coauthors YC, SA, NV, and DH) trained by the senior author (FKC). Accuracy of manual segmentation by these graders were independently verified by the senior author. Most of the segmentation errors were located in regions of RPE loss, because the HEYEX software cannot reliably identify the Bruch's membrane in the absence of an RPE layer. The errors were particularly prominent if there was concomitant choroidal atrophy resulting in Bruch's membrane discontinuity.

**Figure 1.**
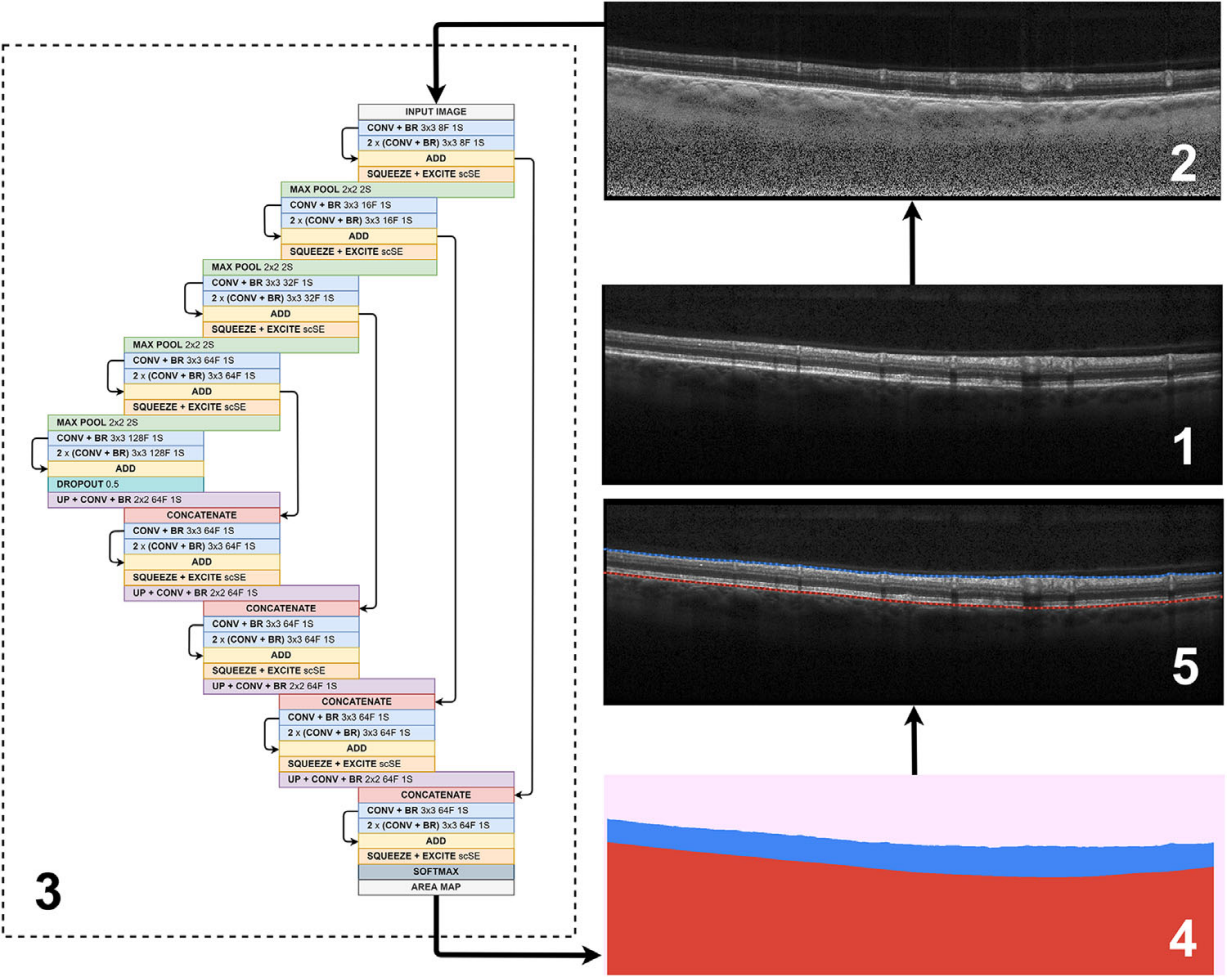
Overview of the proposed method, including (1) an example raw OCT image, (2) corresponding contrast enhanced image, (3) neural network architecture with 4 pooling layers incorporating squeeze and excite blocks, (4) example layer probability map (*pink*, vitreous + padding; *blue*, retina; *red*, choroid + sclera) and, (5) example of OCT image with boundary predictions marked (*solid lines*, truths; *dotted lines*, predictions; *blue lines*, ILM; *red lines*, RPE). For the neural network architecture, #F, filters, #S, stride, BR, batch normalization + ReLU activation, while all convolutional layer inputs are padded such that input size is equal to output size.

A total of 177 OCT volume scan sets from 44 eyes of 22 Stargardt patients were exported via the XML software provided by Heidelberg Engineering (HEYEX-XML). Each scan is grayscale and measures 1536 pixels wide × 496 pixels deep with a transversal resolution 5.7 µm per pixel wide, and axial resolution of 3.9 µm per pixel deep, yielding approximate physical dimensions of 8.8 × 1.9 mm per scan. Examples of a typical OCT scan in Stargardt disease, its automated segmentation and adjusted manual segmentation are shown in Figure 1.

For the purposes of this study, each volume scan set was marked as “usable” based on an inspection of the images and boundary truths. For training purposes and for evaluating boundary error performance, a subset of this usable data was used, including four volumes (the two baseline and latest two follow-up volumes) for each participant (where possible) in an effort to maximize the diversity in the data (image change in the OCT features) as a result of disease progression. The decision to use an equal number of volumes for each participant was taken so that a balanced training dataset could be constructed without bias toward any particular participant. This subset of data was subsequently cleaned using custom software by manually marking a start and an end point inside of which to retain the original image pixels and the provided boundary positions. Outside of this range, the image pixels were zeroed and cropped and the boundary positions were repeated and flattened to the edges of the image. This cleaning and cropping was performed owing to the small region of invalid segmentations of partially truncated images that were commonly present at the lateral extremities of the OCT images. These cleaned data helped to facilitate the training process both so that the method would not learn in an erroneous fashion and so that it may be tested in a fair manner when comparing boundary errors across the entire scan width. During this cleaning process, a small percentage of images (approximately 2.3%) were discarded owing to severe image truncation precluding retinal layer segmentations. For the analysis of thickness and volume metrics, volume data for all tested participants were used. Note that each of these datasets was not cleaned before running this part of the analysis. This information included a total of 7845 images from 129 volumes, with a variable number of volumes per participant.

### Training, Validation, and Testing Sets

Patient images were divided into training (10 subjects), validation (2 subjects) and testing (10 subjects) cohorts with each patient's images used only for one of the three purposes. Early and late disease processes were equally represented in each cohort by (1) calculating the macular volume using the baseline (earliest) scan set, (2) using the median macular volume as a threshold to separate participants into a high- and a low-volume group, and (3) assigning an equal number of each type of patient into the three cohorts. A total of 10 participants (40 volumes) were used for training, 2 participants (8 volumes) were used for validation and 10 participants (39 volumes, with 1 participant only possessing 3 volumes) were used for testing. Note that an individual participant's volumes were used only for training or only for testing, not both. Volumes for an individual participant were not split across the different sets. This cohorting was done to avoid bias in the performance and to obtain the most accurate possible representation of the method's generalizability to new unseen participants. A k-fold approach is used for the training and validation sets with 6 folds used. For each fold, the training set is constructed to contain 10 participants (5 low and 5 high macular volume scan sets), with the validation set containing 2 participants (1 low and 1 high macular volume scan sets). In this way, OCT volume scan sets from all participants were used at least once within the validation set and the balance of low and high macular volume participants is retained for each set, within each fold. A summary of the data split is given in Table 1.

### Preprocessing and Augmentation

The effect of image processing is considered in this study for its effect on performance. Here, a contrast enhancement method proposed by Girard et al.^42^ was examined with emphasis placed on the RPE boundary or Bruch's membrane. This approach is similar to previous studies that have reported related segmentation performance improvements using a similar technique.^20^^,^^22^ An example of the application of the Girard filter is shown in Figure 1. To boost the diversity within the dataset, data augmentation methods were also used. To achieve this, when a sample is presented to the network it is either (each has a 25% chance):

• Unchanged (original image)
• Flipped (left to right/right to left)
• Noisy (Gaussian noise added to each pixel)
• Flipped and noisy

Left–right flips were used because it emulates the conversion of an OCT image from left eye to right and vice versa, and noise was added as a form of regularization and to encourage the methods robustness to varying image quality.^43^ Gaussian noise is added using a variance randomly selected between 250 and 1000, each time a sample is presented. To support all tested neural network architectures, 16 pixels of zero padding were applied to the top of each image.

### Network Architecture and Training

A neural network is used here as the core of the machine learning method. To identify the retinal boundary positions, this network is trained for semantic segmentation to distinguish the three regions separated by these boundaries: the vitreous, the retina, and the sclera. The architecture of this network is inspired by the U-Net,^44^ which is commonly used as the basis for semantic segmentation networks. We have developed this fully semantic network and graph search (FS-GS) in our previous work and have previously demonstrated its application to retinal and choroidal segmentation in images with no pathologic changes.^22^ Additionally, we have highlighted the ability of training such a network to be noise resilient when provided with OCT images of poor quality.^43^ As was the case in our previous studies, eight filters were used in the initial set of convolution blocks with this doubled after each subsequent pooling layer. Each layer consists of three convolutional blocks with a residual connection added between the first and last convolutional blocks by adding their outputs. A 50% dropout is used at the bottleneck for regularization. The Adam optimizer,^45^ with default parameters was used to train the network for 100 epochs by minimizing Dice loss. Afterward, the model with best validation accuracy (highest Dice overlap) was chosen for evaluation. A batch size of three was used for training with all samples randomly shuffled in each epoch. To facilitate implementation of the method a copy of the source code can be found online (https://github.com/jakugel/oct-stargardtretina-seg).

We explore two extensions to the network architecture: (1) varying the number of pooling layers between four and five and (2) incorporating squeeze and excitation blocks.^46^^,^^47^ The motivation for using an additional pooling layer is that there is a greater amount of context available to the network. Indeed, the effective receptive field size of a four-layer variant of our network is 202 × 202, which is increased to 410 × 410 by using an additional pooling layer. Note that we compute these effective receptive field sizes using the general formula provided by Venhuizen et al.^24^ The advantage of additional context is that feature extraction may be performed on a more global level.

The idea of the second proposed extension, using squeeze and excitation blocks, is to increase the representational power of the network. First, we provide some background on the concepts of feature maps and the terms “spatial” and “channel.” Put simply, the feature maps contain information (or features) learnt by the network. For an OCT image, this information (these features) might include (but is not limited to) layer boundaries, layer areas, artifacts, speckle noise, blood vessel shadows, or other structures. Each feature map may contain a different subset of features. We also define the term “spatial” here to refer to the spatial dimensions (i.e., width and height) of each feature map and using the term “channel” to refer to an individual feature map. Squeeze and excitation blocks operate by reweighting the importance of the feature maps (at any given layer output in the network) to place more emphasis on (1) the more important and relevant feature maps (channels) and/or (2) the more important and relevant spatial locations within the feature maps. We provide some examples. In the first case, feature maps that contain information related to the layer positions are likely to be more relevant and important for this segmentation problem and could be weighted higher. Similarly in the second case, spatial locations in the feature maps that correspond with transitions between layers (the boundaries) are particularly relevant for this segmentation problem and could also be weighted higher. By placing such blocks at the output of each level of the network, the learned feature maps are dynamically recalibrated both spatially and channel-wise, producing a set of reweighted feature maps that are likely to be more meaningful for this particular application and thus may help to improve segmentation performance. There are three variants of these blocks and, in our network, we use the concurrent spatial and channel squeeze and excitation block variant. Further details about the concurrent spatial and channel squeeze and excitation block as well as the other two block variants are provided in Supplementary Figure S1 and the accompanying text.

Rather than using a single training and validation set, the six folds may be used to each separately train a network to be evaluated on an identical testing set. Here, two methods were used:

1) Average the boundary errors for the six networks (average); and
2) Majority vote on each boundary probability map with subsequent boundary delineation performed on the single map (ensemble).

Owing to inherent randomness in the initial weights of the neural networks, as well as the order of presentation of samples from shuffling, each experiment was performed five times with the results averaged.

### Boundary Delineation and Graph Search

The GS method used for boundary delineation is similar to that used in a number of previous studies.^12^^,^^18^^–^^20^^,^^22^ Using a trained network, layer probability maps for test images may be obtained. To obtain boundary probability maps, edge detection is then performed with a boundary probability map formed for each boundary. An acyclic directed graph is then constructed with each pixel corresponding to a vertex. All vertices are connected left to right to their three rightmost immediate neighbors (horizontally, diagonally up, and diagonally down). The weights of the edges are computed using the following formula:

$$\begin{array}{c} w_{sd}=2-\left(p_{s}+p_{d}\right) \end{array}$$

where *p_s_* and *p_d_* are the probabilities of the source and destination vertices respectively. Using Dijkstra's shortest path algorithm, a graph search is then performed to find the shortest path of the graph using predetermined image locations, the start (top left corner) and the end (bottom right corner). This shortest path corresponds with the predicted boundary location. To gauge performance, the predicted boundary location is compared with the ground truth, with the mean absolute error (MAE) and mean error in pixels computed.

### Comparison of Methods

There has been a vast array of previous methods proposed for retinal segmentation, so the usefulness of the proposed method should be validated by comparing with these where possible. Here, three other methods are considered. The first is a patch-based machine learning method.^18^^–^^20^^,^^21^^,^^22^ For this, we use the Cifar CNN architecture and a set of patch classes (single background) proposed by Fang et al.^18^ and a graph search method and 64 × 32 patch size used by Kugelman et al.^20^ Here, patches are constructed using the same set of images as the proposed method, with one patch for each class sampled from every column where segmentations are present. The other two methods for comparison, not based on machine learning, are publicly available tools including the OCTExplorer tool (part of the IOWA Reference Algorithms [Retinal Image Analysis Lab, Iowa Institute for Biomedical Imaging, Iowa City, IA]),^14^^,^^48^^,^^49^ and the AURA tool for retinal layer segmentation.^17^

## Results

For the proposed semantic segmentation and graph search method, denoted FS-GS, five variants of the overall model are considered, including the effect of contrast enhancement, number of pooling layers, use of squeeze and excitation blocks, and whether augmentations were used. Each method was run five times for each of six folds with the trained networks evaluated by calculating the MAE and mean error for each boundary. The mean results across the five runs for both the average fold performance and the ensemble performance are summarized in Table 2 (MAE) and Table 3 (mean error). The Dice overlap (percent) for each method, for the network predictions (pregraph search) were also computed for both the retina as well as the overall value. These values are provided in Supplementary Table S1.

**Table 1.** Summary of Training, Validation, and Testing Sets Used

Set	No. of Images^†^	No. of Participants	No. of Volumes
Training	2424–2429	10	40
Validation	483–488	2	8
Testing (subset, all)	2259 (7845)	10	39 (129)
Total (subset, all)	5171 (10,762)	22	87 (177)

† The number of images for training and validation varies very slightly between folds owing to the handful of truncated scans that were discarded. For evaluating boundary error performance, the subset of cleaned test data is used (2259 images), whereas all testing data (uncleaned) is used for volume and thickness calculations (7845 images).

**Table 2.** Boundary MAE Results for Inner and Outer Retinal Boundaries

	Average		Ensemble	
Method	ILM MAE (SD) [px]	RPE MAE (SD) [px]	ILM MAE (SD) [px]	RPE MAE (SD) [px]
ON 4	0.32 (0.68)	1.37 (2.42)	0.23 (0.20)	1.22 (1.74)
OFF 4	0.36 (1.10)	1.47 (3.03)	0.25 (0.45)	1.37 (2.54)
ON 5	0.32 (0.81)	1.39 (2.96)	0.23 (0.30)	1.25 (2.28)
ON 4 scSE	0.35 (1.28)	1.31 (2.15)	0.23 (0.23)	1.12 (1.41)
ON 4 scSE NA	0.34 (0.88)	1.41 (2.50)	0.22 (0.24)	1.17 (1.60)

Each method was run five times with the average error and standard deviation across the five runs presented here. 4/5, number of pooling layers used; NA, no augmentations were used; ON/OFF, whether contrast enhancement was used; px, pixels; scSE, squeeze + excitation blocks were incorporated.

**Table 3.** Boundary Mean Error Results for Inner and Outer Retinal Boundaries

	Average		Ensemble	
Method	ILM ME (SD) [px]	RPE ME (SD) [px]	ILM ME (SD) [px]	RPE ME (SD) [px]
ON 4	0.02 (1.32)	0.15 (2.15)	‒0.02 (0.20)	0.14 (1.79)
OFF 4	0.14 (1.08)	0.24 (2.84)	0.05 (0.51)	0.18 (2.96)
ON 5	0.05 (0.79)	0.23 (2.82)	‒0.02 (0.32)	0.21 (2.62)
ON 4 scSE	0.01 (1.26)	0.15 (2.16)	‒0.02 (0.20)	0.17 (1.46)
ON 4 scSE NA	0.09 (0.86)	0.16 (2.51)	‒0.01 (0.22)	0.10 (1.61)

Each method was run five times with the average error and standard deviation across the five runs presented here. Positive values indicate that predictions are lower on the image than the corresponding truths. 4/5, number of pooling layers used; ME, mean error; NA, no augmentations were used; ON/OFF, whether contrast enhancement was used; px, pixels; scSE, squeeze + excitation blocks were incorporated.

**Figure 2.**
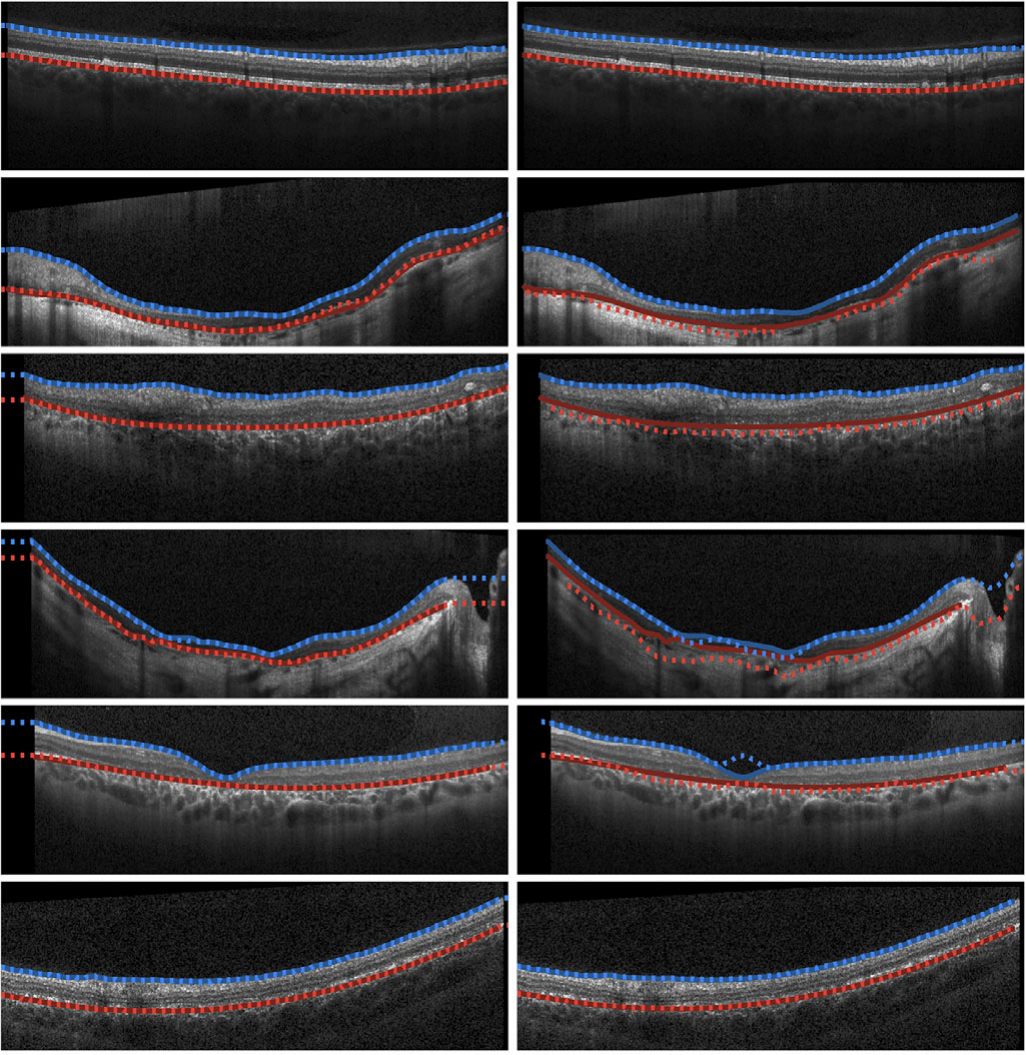
Example segmentations of Stargardt OCT images from FS-GS (the proposed ML-based semantic segmentation) method (*left*) and those provided by OCTExplorer (*right*). *Blue*, ILM; *Red*, RPE/Bruch's membrane. *Solid lines* indicate the ground truth boundary locations and the *dotted lines* correspond with the predicted locations.

We also compare the best performing method to a range of other approaches with the boundary error (MAE) results summarized in Table 4. For further reference, the Spectralis segmentation algorithm (Heidelberg Eye Explorer version 1.9.14.0) is also evaluated against the ground truth corrected data. For these, we note that some methods failed to provide segmentations for part or whole of some scans and volumes. Because of this, these parts are not included in error calculations for those individual methods. In particular, AURA failed to segment 7 of 39 (18%) of the testing volumes, and OCTExplorer returned no predictions for approximately 1.7% of the total image columns used for comparison. Figure 2 provides some example segmentations for both the proposed method and OCTExplorer for a variety of scans. Supplementary Figures S2 and S3 provide the same comparison with the AURA method and the patch-based machine learning method respectively.

**Table 4.** Comparison of Various Methods with the Best Performing Semantic Segmentation Method

Method	ILM MAE (SD) [px]	RPE MAE (SD) [px]
FS-GS [Semantic ON 4 scSE] (ensemble)	0.23 (0.23)	1.12 (1.41)
Patch-based 64 × 32 (ensemble)	0.26 (0.17)	1.15 (1.13)
OCTExplorer^^^ [54]	1.32 (1.41)	4.95 (5.33)
AURA^*^ [20]	1.26 (0.92)	7.17 (6.87)
Spectralis (automatic)	1.07 (1.63)	6.50 (3.27)

* Some volume segmentations failed (18% of all testing volumes) and could not be included in error calculations.

^ Sections of some scans (1.7% of compared columns) returned undefined segmentations. px, pixels; scSE, squeeze + excitation blocks were incorporated.

**Figure 3.**
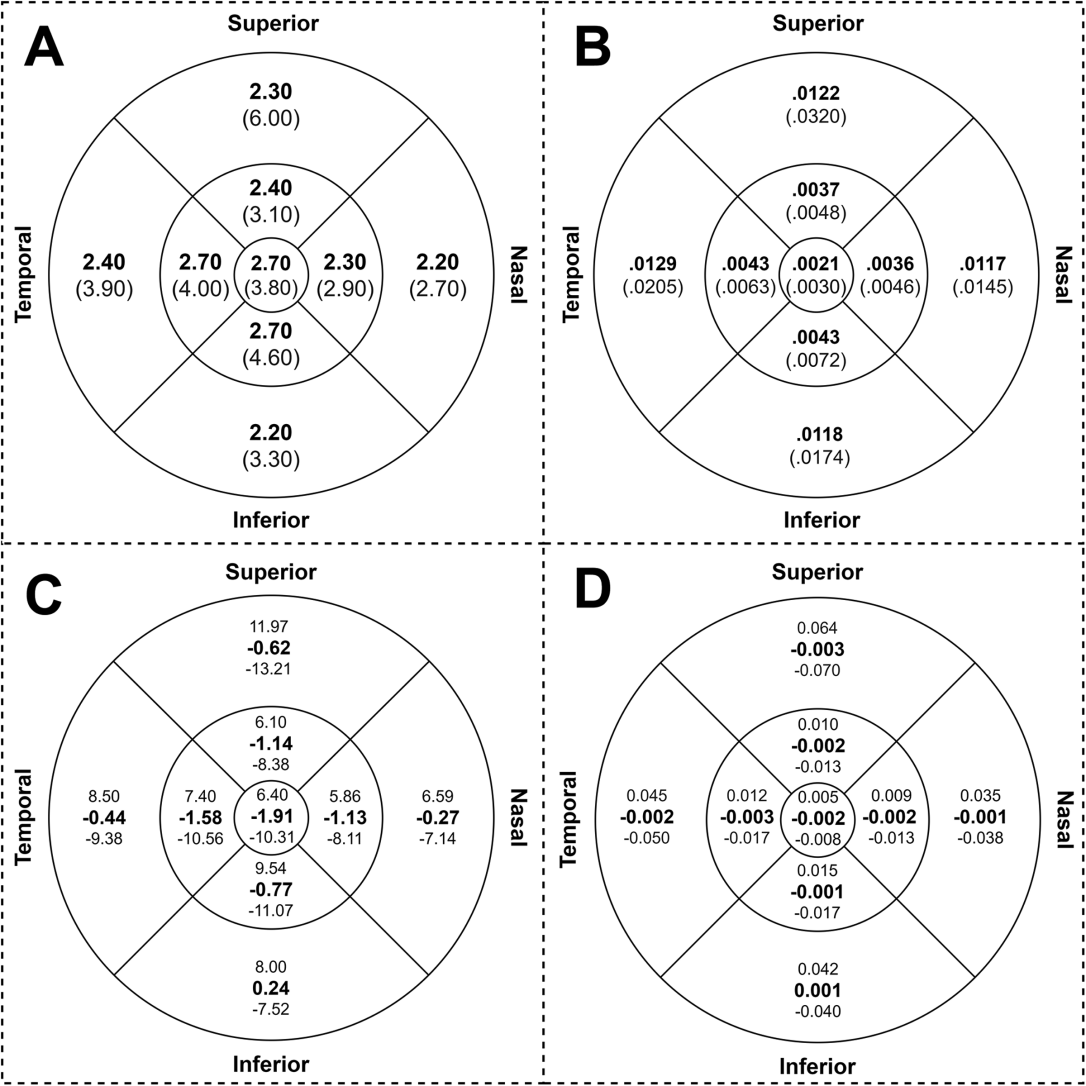
Mean absolute difference (**in bold**) and standard deviation in (parentheses) of the thickness (in µm) (subplot A) and volume in cubic millimeters (subplot B). Mean (**in bold**) and limits of agreement (+1.96 SD above, –1.96 SD below) from Bland–Altman analysis between FS-GS and the ground truth for each subfield for thickness (in µm) (subplot C) and volume cubic millimeters (subplot D). Measurements are performed across the entire testing dataset for all nine Early Treatment of Diabetic Retinopathy Study subfields across the central 6 mm of all 129 testing volumes. Here the circles (from inner to outer, respectively) represent 1, 3, and 6 mm in diameter.

We also quantify the error (mean absolute difference) for both thickness and volume for all nine Early Treatment of Diabetic Retinopathy Study subfields across the central 6-mm diameter in all testing volumes. This included all 129 volume scan sets from the testing participants. The mean absolute differences between the retinal thickness and volumes generated by FS-GS and the ground truth were 2.10 µm and 0.059 mm^3^, respectively. The detailed results of the analysis for each subfield are summarized in Figure 3 (subplots A and B, respectively). A Bland–Altman analysis was undertaken with respect to both thickness and volume for each subfield as well as for the total thickness and total volume across the central 6-mm diameter zone. For the total thickness, the mean difference between FS-GS and the ground truth was −0.55 µm with limits of agreement of −7.66 µm and +6.56 µm. For the total volume, the mean difference was −0.0156 mm^3^ with limits of agreement of −0.2166 mm^3^ and +0.1855 mm^3^. The respective results for each subfield are summarized in Figure 3 (subplots C and D respectively). Bland–Altman plots for the total central 6-mm diameter zone thickness and volume are provided in Supplementary Figure S4.

## Discussion

In this study, we have developed and evaluated a fully automated method (FS-GS) to segment the inner and outer retinal boundaries in OCT images from patients with Stargardt disease. We have performed a number of experiments to further develop and optimize the neural network architecture and machine learning ensemble processes used as the basis for FS-GS. Compared with existing, publicly available segmentation software, FS-GS performs favorably with a significant improvement in boundary delineation accuracy and a greater level of consistency. Critically, from a clinical point of view, there is a high level of agreement between FS-GS and the ground truth supported by the negligible differences in mean retinal thickness and total macular volume across all Early Treatment of Diabetic Retinopathy Study subfields in the central 6 mm diameter zone.

Our experiment illustrates several key ingredients of success in our proposed automated segmentation method (FS-GS). First, the use of contrast enhancement as a preprocessing step seems to improve the segmentation performance, particularly on the RPE/Bruch's membrane boundary, with a significant decrease in the standard deviation of the error. Importantly, the enhanced performance at the outer boundary does not seem to compromise the accuracy of the segmentation at the ILM boundary. Second, the use of an additional pooling layer in the case of five pooling layers does not appear to have a large effect on performance. Therefore, it is likely that additional context is not needed in the form of a larger effective receptive field (410 × 410 pixels) than what is already provided by the four-layer network variant (202 × 202 pixels). Third, unlike results from other studies,^46^^,^^47^ adding squeeze plus excitation blocks did seem to only slightly improve the segmentation performance for the RPE/Bruch's membrane boundary, with a decrease of 0.10 pixels evident when comparing the respective ensemble models. Fourth, ensembling itself had a noticeable positive impact. In all cases, ensembling led to a lower mean and standard deviation in all boundary errors. Indeed, performance improvements with respect to the MAE were observed on the ILM (approximately 0.10 pixels) and on the RPE (0.10–0.25 pixels) across the board. Fifth, small performance improvements were identified when augmentations were used but, critically, it should be noted that the use of the chosen augmentations does not lead to a decrease in performance. The mean error calculations suggest that the boundary predictions do not exhibit any consistent bias above or below the ILM boundary, with generally small values observed on average. For the RPE/Bruch's membrane, there was a slight trend for the prediction boundary to be located below the ground truth positions, but again these values were small compared with the corresponding MAEs.

Interestingly, the Dice overlap values did not seem to correlate with the boundary errors. For instance, the Dice values between the average and ensemble approaches were very similar across the different evaluated methods, whereas the corresponding boundary errors are notably lower using the ensemble approach. This finding indicates that any differences between the two methods only occur in a very small margin around the boundaries.

A number of existing non–machine learning–based methods were also evaluated for their performance. We demonstrated that mean absolute boundary position errors on both the ILM and the RPE/Bruch's membrane are considerably smaller and much more consistent (lower standard deviations) across scans with the use of FS-GS compared with existing methods. Indeed, a decrease in MAE of approximately one-pixel was observed on the ILM and a decrease of more than three pixels was observed on the RPE/Bruch's membrane compared with these other methods. These differences were significant despite the exclusion of regions of scans and volumes where segmentations failed using existing methods. Indeed, the existing methods appear to be inferior on two counts: (1) the greater and less consistent boundary errors and (2) the failure to segment regions of some scans and volumes. For instance, the second row in Figure 2 shows an example of a central portion of an OCT scan with no predicted boundary locations provided by OCTExplorer. In contrast, FS-GS is constrained to provide predictions for the entire width of each scan. The other examples in Figure 2 show a range of other cases where OCTExplorer provides comparable segmentations (first and last rows) and relatively poor performance compared with the proposed method (middle rows). It seems that the scans exhibiting more advanced retinal and choroidal atrophy are the more difficult cases for the segmentation algorithm. Here, it is logical that these existing methods perform poorly because these algorithms were not originally designed to handle images of pathology of this type. Additionally, we analyzed the level of manual correction that was performed on the automatic segmentation provided by the Heidelberg Spectralis OCT instrument. Again, the segmentation error between the corrected and uncorrected ground truth is high and on a similar level to the other automatic methods, demonstrating that the automatic Spectralis segmentation algorithm used by the instrument is less efficient for this dataset. Overall, it is clear that the performance of these existing methods justifies the need for the improved FS-GS method presented here.

A patch-based machine learning method demonstrated similar results to FS-GS. Here, MAEs were slightly lower in FS-GS but standard deviations were slightly higher. The similarity between the two methods is highlighted by the little difference between the predicted boundaries as illustrated by the example plots in Supplementary Figure S3. However, as has been highlighted in a previous study,^22^ such patch-based methods are significantly more time consuming with respect to evaluation time than FS-GS. Here, a similar semantic segmentation method performed evaluations in about one-half the time when comparing the same patch size and patch classification architecture as used in this study. The chosen patch size of this method is 64 × 32 pixels, which is considerably smaller than the effective receptive field of the proposed network (202 × 202 pixels). This finding once again demonstrates that the context available to the network does not seem to be a limiting factor in the performance of the FS-GS method.

The reproducibility in clinically relevant metrics (retinal thickness and volume in central 6-mm zone) using our FS-GS method was comparable to the repeatability of manual segmentation in diseased retina. The mean absolute and relative differences in retinal thicknesses between FS-GS and the ground truth was 2.10 µm and −0.55 µm, respectively, whereas the coefficient of repeatability of manual segmentation was 4.5 µm in a previous report. Similarly, the mean absolute and relative differences in retinal volume between FS-GS and the ground truth was 0.059 mm^3^ and −0.016 mm^3^, respectively, which was less than the coefficient of repeatability in manual segmentation of 0.164 mm^3^ as reported by Hanumunthadu et al.^6^ However, the limits of agreement were slightly greater than the coefficients of repeatability and the impact of this variability on tracking disease progression rate compared with using ground truth measurements warrants further study.

Although this study is the first attempt to use machine learning for training a segmentation algorithm specific for OCT scans of patients with Stargardt disease, the training set of approximately 2400 B-scans was relatively small and they were from only 10 patients. We tried to diversify the training set by including equal number of eyes with low and high macular volumes so that the algorithm can recognize the varied morphology of the RPE/Bruch's membrane boundary in different stages of the disease. However, 10 patients cannot represent the entire spectrum of Stargardt disease phenotype and disease severity. The training set consisted of scans from the Heidelberg device and, therefore, the reproducibility reported for a testing set of OCT images from this device is likely to be different for OCT scans from other spectral domain or swept source OCT devices using our FS-GS method. We only examined the accuracy of segmentation at the level of the ILM and RPE/Bruch's membrane. Previous authors have attempted to segment internal layers of the retina, but these boundaries are often difficult to distinguish in severe retinal degeneration without a direct correlation with histology.^39^ Hence, further training of FS-GS is required to provide sublayer segmentation and volume measurement. Similarly, further training may also be required to adapt FS-GS to other pathologies and differing levels of OCT image quality,^43^ as well as images acquired using other scanning parameters (e.g., scan averaging, enhanced depth imaging).

Although the focus of this work was the segmentation of Stargardt's images, the translational impact of FS-GS to nonpathologic images is also an important consideration. To obtain a better idea of this factor, we used FS-GS to perform segmentation on two volumetric image sets from healthy participants. Across approximately 110 total images (OCT B-scans) the segmentation errors were very low (ILM MAE of 0.18 pixels and RPE MAE of 0.25 pixels). This result demonstrates that good performance can be maintained for healthy images. We note that the reason behind this finding is that early stage pathologic images, used to train the model, still closely resemble those of healthy images.

In conclusion, we have demonstrated that our proposed method, FS-GS, exhibits promising performance for the segmentation of OCT images in Stargardt disease. Using such a method in clinical practice may allow for a more efficient segmentation process and reduce the burden of OCT interpretation by eliminating the need for manual correction of software errors from existing methods. Future development of machine learning methods should ideally be agnostic to the type of OCT instrument and provide segmentation of the internal layers of the retina.

## Supplementary Material

Supplement 1

Supplement 2

Supplement 3

Supplement 4

Supplement 5

Supplement 6

## References

[1] Liew G, Michaelides M, Bunce C A comparison of the causes of blindness certifications in England and Wales in working age adults (16–64 years), 1999–2000 with 2009–2010. *BMJ Open*. 2014; 4: e004015. 10.1136/bmjopen-2013-004015 PMC3927710 24525390

[2] Crewe J, Morgan WH, Morlet N, et al. Prevalence of blindness in Western Australia: a population study using capture and recapture techniques. *Br J Ophthalmol*. 2012; 96: 478–481. 22096138 10.1136/bjophthalmol-2011-300908

[3] Alshareef RA, Goud A, Mikhail M, et al. Segmentation errors in macular ganglion cell analysis as determined by optical coherence tomography in eyes with macular pathology. *Int J Retina Vitreous*. 2017; 3: 25. 28725485 10.1186/s40942-017-0078-7 PMC5513039

[4] Aojula A, Mollan SP, Horsburgh J, et al. Segmentation error in spectral domain optical coherence tomography measures of the retinal nerve fibre layer thickness in idiopathic intracranial hypertension. *BMC Ophthalmol*. 2017; 17: 257. 10.1186/s12886-017-0652-7 PMC6389234 29298687

[5] Patel PJ, Chen FK, da Cruz L, Tufail A Segmentation error in stratus optical coherence tomography for neovascular age-related macular degeneration. *Invest Ophthalmol Vis Sci*. 2009; 50: 399–404. 18676631 10.1167/iovs.08-1697

[6] Hanumunthadu D, Wang JP, Chen W, et al. Impact of retinal pigment epithelium pathology on spectral-domain optical coherence tomography derived macular thickness and volume metrics and their intersession repeatability. *Clin Exp Ophthalmol*. 2017; 45: 270–279. 28052542 10.1111/ceo.12868

[7] Liao DS, Grossi FV, El Mehdi D, et al. Complement C3 inhibitor pegcetacoplan for geographic atrophy secondary to age-related macular degeneration: a randomized phase 2 trial. *Ophthalmology*. 2020; 127: 186–195. 31474439 10.1016/j.ophtha.2019.07.011

[8] Cremers FPM, Lee W, Collin RWJ, Allikmets R Clinical spectrum, genetic complexity and therapeutic approaches for retinal disease caused by ABCA4 mutations. *Prog Retin Eye Res*. 2020; 9: 100861. 10.1016/j.preteyeres.2020.100861 PMC7544654 32278709

[9] Baghaie A, Yu Z, D'Souza RM State-of-the-art in retinal optical coherence tomography analysis. *Quant Imaging Med Surg*. 2015; 5: 603–617. 26435924 10.3978/j.issn.2223-4292.2015.07.02 PMC4559975

[10] DeBuc DC. A review of algorithms for segmentation of retinal image data using optical coherence tomography. Ho PG (ed.) *Image Segmentation*. London: InTech; 2011: 15–54.

[11] González-López A, de Moura J, Novo J, Ortega M, Penedo MG Robust segmentation of retinal layers in optical coherence tomography images based on a multistage active contour model. *Heliyon*. 2019; 5: e01271. 30891515 10.1016/j.heliyon.2019.e01271 PMC6401526

[12] Chiu SJ, Li XT, Nicholas P, Toth CA, Izatt JA, Farsiu S Automatic segmentation of seven retinal layers in SDOCT images congruent with expert manual segmentation. *Opt Express*. 2010; 18: 19413–19428. 20940837 10.1364/OE.18.019413 PMC3408910

[13] Chiu SJ, Allingham MJ, Mettu PS, Cousins SW, Izatt JA, Farsiu S Kernel regression based segmentation of optical coherence tomography images with diabetic macular edema. *Biomed Opt Express*. 2015; 6: 1172–1194. 25909003 10.1364/BOE.6.001172 PMC4399658

[14] Li K, Wu X, Chen DZ, Sonka M Optimal surface segmentation in volumetric images – a graph – theoretic approach. *IEEE Trans Pattern Anal Mach Intell*. 2006; 28: 119–134. 16402624 10.1109/TPAMI.2006.19 PMC2646122

[15] Vermeer K, V der Schoot J, Lemij H, De Boer J Automated segmentation by pixel classification of retinal layers in ophthalmic OCT images. *Biomed Opt Express*. 2011; 2: 1743–1756. 21698034 10.1364/BOE.2.001743 PMC3114239

[16] Srinivasan PP, Heflin SJ, Izatt JA, Arshavsky VY, Farsiu S Automatic segmentation of up to ten layer boundaries in SD-OCT images of the mouse retina with and without missing layers due to pathology. *Biomed Opt Express*. 2014; 5: 348–365. 24575332 10.1364/BOE.5.000348 PMC3920868

[17] Lang A, Carass A, Hauser M, et al. Retinal layer segmentation of macular OCT images using boundary classification. *Biomed Opt Express*. 2013; 4: 1133–1152. 23847738 10.1364/BOE.4.001133 PMC3704094

[18] Fang L, Cunefare D, Wang C, Guymer RH, Li S, Farsiu S Automatic segmentation of nine retinal layer boundaries in OCT images of non-exudative AMD patients using deep learning and graph search. *Biomed Opt Express*. 2017; 8: 2732–2744. 28663902 10.1364/BOE.8.002732 PMC5480509

[19] Hamwood J, Alonso-Caneiro D, Read SA, Vincent SJ, Collins MJ Effect of patch size and network architecture on a convolutional neural network approach for automatic segmentation of OCT retinal layers. *Biomed Opt Express*. 2018; 9: 3049–3066. 29984082 10.1364/BOE.9.003049 PMC6033561

[20] Kugelman J, Alonso-Caneiro D, Read SA, Vincent SJ, Collins MJ Automatic segmentation of OCT retinal boundaries using recurrent neural networks and graph search. *Biomed Opt Express*. 2018; 9: 5759–5777. 30460160 10.1364/BOE.9.005759 PMC6238930

[21] Hu K, Shen B, Zhang Y, Cao C, Xiao F, Gao X Automatic segmentation of retinal layer boundaries in OCT images using multiscale convolutional neural network and graph search. *Neurocomputing*. 2019; 365: 302–313.

[22] Kugelman J, Alonso-Caneiro D, Read SA, et al. Automatic choroidal segmentation in OCT images using supervised deep learning methods. *Sci Rep*. 2019; 9: 13298. 31527630 10.1038/s41598-019-49816-4 PMC6746702

[23] Roy AG, Conjeti S, Karri SPK, et al. ReLayNet: retinal layer and fluid segmentation of macular optical coherence tomography using fully convolutional network. *Biomed Opt Express*. 2017; 8: 3627–3642. 28856040 10.1364/BOE.8.003627 PMC5560830

[24] Venhuizen FG, van Ginneken B, Liefers B, et al. Robust total retina thickness segmentation in optical coherence tomography images using convolutional neural networks. *Biomed Opt Express*. 2017; 8: 3292–3316. 28717568 10.1364/BOE.8.003292 PMC5508829

[25] Pekala M, Joshi N, Alvin Liu TY, Bressler NM, Cabrera DeBuc D, Burlina P Deep learning based retinal OCT segmentation. *Comput Biol Med*. 2019; 114: 103445. 31561100 10.1016/j.compbiomed.2019.103445

[26] He Y, Carass A, Liu Y, et al. Deep learning based topology guaranteed surface and MME segmentation of multiple sclerosis subjects from retinal OCT. *Biomed Opt Express*. 2019; 10: 5042–5058. 31646029 10.1364/BOE.10.005042 PMC6788619

[27] Shah A, Abramoff M, Wu X Simultaneous multiple surface segmentation using deep learning. Jorge Cardoso M, Arbel T, Carneiro G, et al. (eds.) *Deep Learning in Medical Image Analysis and Multimodal Learning for Clinical Decision Support*. New York: Springer; 2017: 3–11.

[28] Xu Y, Yan K, Kim J,, et al. Dual-stage deep learning framework for pigment epithelium detachment segmentation in polypoidal choroidal vasculopathy. *Biomed Opt Express*. 2017; 8: 4061–4076. 28966847 10.1364/BOE.8.004061 PMC5611923

[29] Loo J, Fang L, Cunefare D, Jaffe GJ, Farsiu S Deep longitudinal transfer learning-based automatic segmentation of photoreceptor ellipsoid zone defects on optical coherence tomography images of macular telangiectasia type 2. *Biomed Opt Express*. 2018; 9: 2681–2698. 30258683 10.1364/BOE.9.002681 PMC6154208

[30] Wang J, Wang Z, Li F, Qu G, Qiao Y, Hairong L, Zhang X Joint retina segmentation and classification for early glaucoma diagnosis. *Biomed Opt Express*. 2019; 10: 2639–2656. 31149385 10.1364/BOE.10.002639 PMC6524599

[31] Wang Y, Galles D, Klein M, Locke KG, Birch DG Application of a deep machine learning model for automatic measurement of EZ width in SD-OCT images of RP. *Trans Vis Sci Technol*. 2020; 9: 15. 10.1167/tvst.9.2.15 PMC7395669 32818077

[32] Wong BM, Cheng RW, Mandelcorn ED,, et al. Validation of optical coherence tomography retinal segmentation in neurodegenerative disease. *Trans Vis Sci Technol*. 2019; 8: 6. 10.1167/tvst.8.5.6 PMC6753973 31588371

[33] Querques G, Leveziel N, Benhamou N, Voigt M, Soubrane G, Souied EH Analysis of retinal flecks in fundus flavimaculatus using optical coherence tomography. *Br J Ophthalmol*. 2006; 90: 1157–1162. 16754647 10.1136/bjo.2006.094136 PMC1857370

[34] Yeoh J, Rahman W, Chen F, et al. Choroidal imaging in inherited retinal disease using the technique of enhanced depth imaging optical coherence tomography. *Graefes Arch Clin Exp Ophthalmol*. 2010; 248: 1719–1728. 20640437 10.1007/s00417-010-1437-3

[35] Park SP, Chang S, Allikmets R, et al. Disruption in Bruch membrane in patients with Stargardt disease. *Ophthalmic Genet*. 2012; 33: 49–52. 22060670 10.3109/13816810.2011.628358 PMC4407800

[36] Adhi M, Read SP, Ferrara D, Weber M, Duker JS, Waheed NK Morphology and vascular layers of the choroid in Stargardt disease analysed using spectral-domain optical coherence tomography. *Am J Ophthalmol*. 2015; 160: 1276–1284. 26314663 10.1016/j.ajo.2015.08.025

[37] Kong X, Ho A, Munoz B, et al. Reproducibility of measurements of retinal structural parameters using optical coherence tomography in Stargardt disease. *Trans Vis Sci Technol*. 2019; 8: 46. 10.1167/tvst.8.3.46 PMC6590092 31259091

[38] Strauss RW, Muñoz B, Wolfson Y, et al. Assessment of estimated retinal atrophy progression in Stargardt macular dystrophy using spectral-domain optical coherence tomography. *Br J Ophthalmol*. 2015; 100: 956–962. 26568636 10.1136/bjophthalmol-2015-307035 PMC4941136

[39] Velaga SB, Nittala MG, Jenkins D, et al. Impact of segmentation density on spectral domain optical coherence tomography assessment in Stargardt disease. *Graefes Arch Clin Exp Ophthalmol*. 2019; 257: 549–556. 30613916 10.1007/s00417-018-04229-3

[40] Davidson B, Kalitzeos A, Carroll J, et al. Automatic cone photoreceptor localisation in healthy and Stargardt afflicted retinas using deep learning. *Sci Rep*. 2018; 8: 7911. 29784939 10.1038/s41598-018-26350-3 PMC5962538

[41] Alonso-Caneiro D, Read SA, Vincent SJ, Collins MJ, Wojtkowski M Tissue thickness calculation in optical coherence tomography. *Biomed Opt Express*. 2016; 7: 629–645. 26977367 10.1364/BOE.7.000629 PMC4771476

[42] Girard MJ, Strouthidis NG, Ethier CR, Mari JM Shadow removal and contrast enhancement in optical coherence tomography images of the human optic nerve head. *Invest Ophthalmol Vis Sci*. 2011; 52: 7738–7748. 21551412 10.1167/iovs.10-6925

[43] Kugelman J, Alonso-Caneiro D, Read SA, Vincent SJ, Chen FK, Collins MJ Effect of altered OCT image quality on deep learning boundary segmentation. *IEEE Access* 2020; 8: 43537–43553.

[44] Ronneberger O, Fischer P, Brox T U-Net: convolutional networks for biomedical image segmentation. 2015. arxiv:1505.04597.

[45] Kingma DP, & Ba J. Adam: A method for stochastic optimization. 2014. arXiv preprint arXiv:1412.6980.

[46] Hu J, Shen L, Sun G Squeeze-and-excitation networks. In Proceedings of the IEEE conference on computer vision and pattern recognition. 2018 (pp. 7132–7141.

[47] Roy AG, Navab N, Wachinger C Recalibrating fully convolutional networks with spatial and channel ‘squeeze & excitation’ blocks. 2018. arXiv:1808.08127. 10.1109/TMI.2018.2867261 30716024

[48] Abramoff MD, Garvin M, Sonka M Retinal imaging and image analysis. *IEEE Rev Biomed Eng*. 2010; 3: 169–208. 22275207 10.1109/RBME.2010.2084567 PMC3131209

[49] Garvin MK, Abramoff MD, Wu X, Burns TK, Russell SR, Sonka M Automated 3-D intraretinal layer segmentation of macular spectral-domain optical coherence tomography images. *IEEE Trans Med Imaging*. 2009; 9: 1436–1447. 10.1109/TMI.2009.2016958 PMC2911837 19278927

