# Supplementary material for: Retinal Boundary Segmentation in Stargardt Disease Optical Coherence Tomography Images Using Automated Deep Learning
Source: Transl Vis Sci Technol. 2020 Oct 13;9(11):12. doi: 10.1167/tvst.9.11.12 (PMC7581491; doi:10.1167/tvst.9.11.12)
Supplement: Supplement 1 [file tvst-9-11-12_s001.pdf]

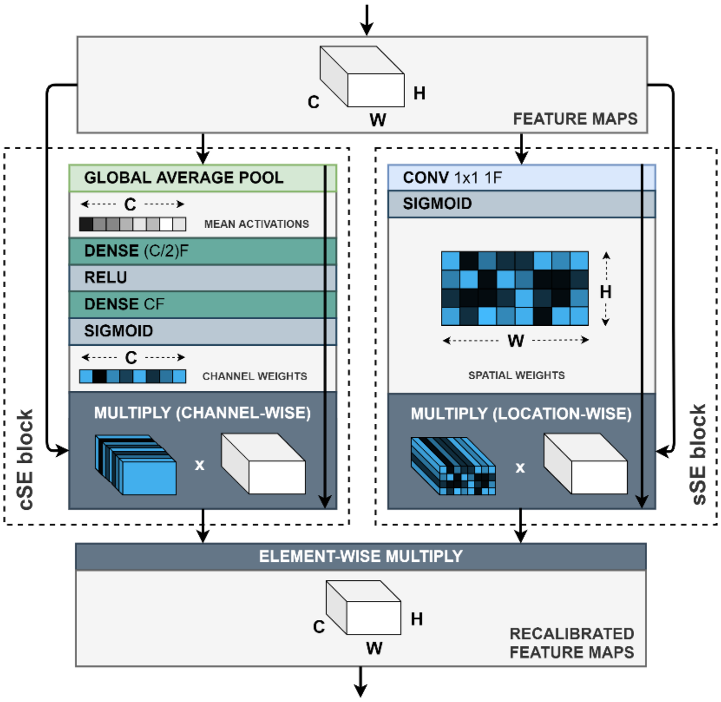

Supplementary Fig 1: Overview of the concurrent spatial and channel squeeze and excitation block (scSE). #F: number of filters, H: height, W: width, C: number of channels. Solid lines indicate information flow.
