# Supplementary material for: Retinal Boundary Segmentation in Stargardt Disease Optical Coherence Tomography Images Using Automated Deep Learning
Source: Transl Vis Sci Technol. 2020 Oct 13;9(11):12. doi: 10.1167/tvst.9.11.12 (PMC7581491; doi:10.1167/tvst.9.11.12)
Supplement: Supplement 3 [file tvst-9-11-12_s003.pdf]

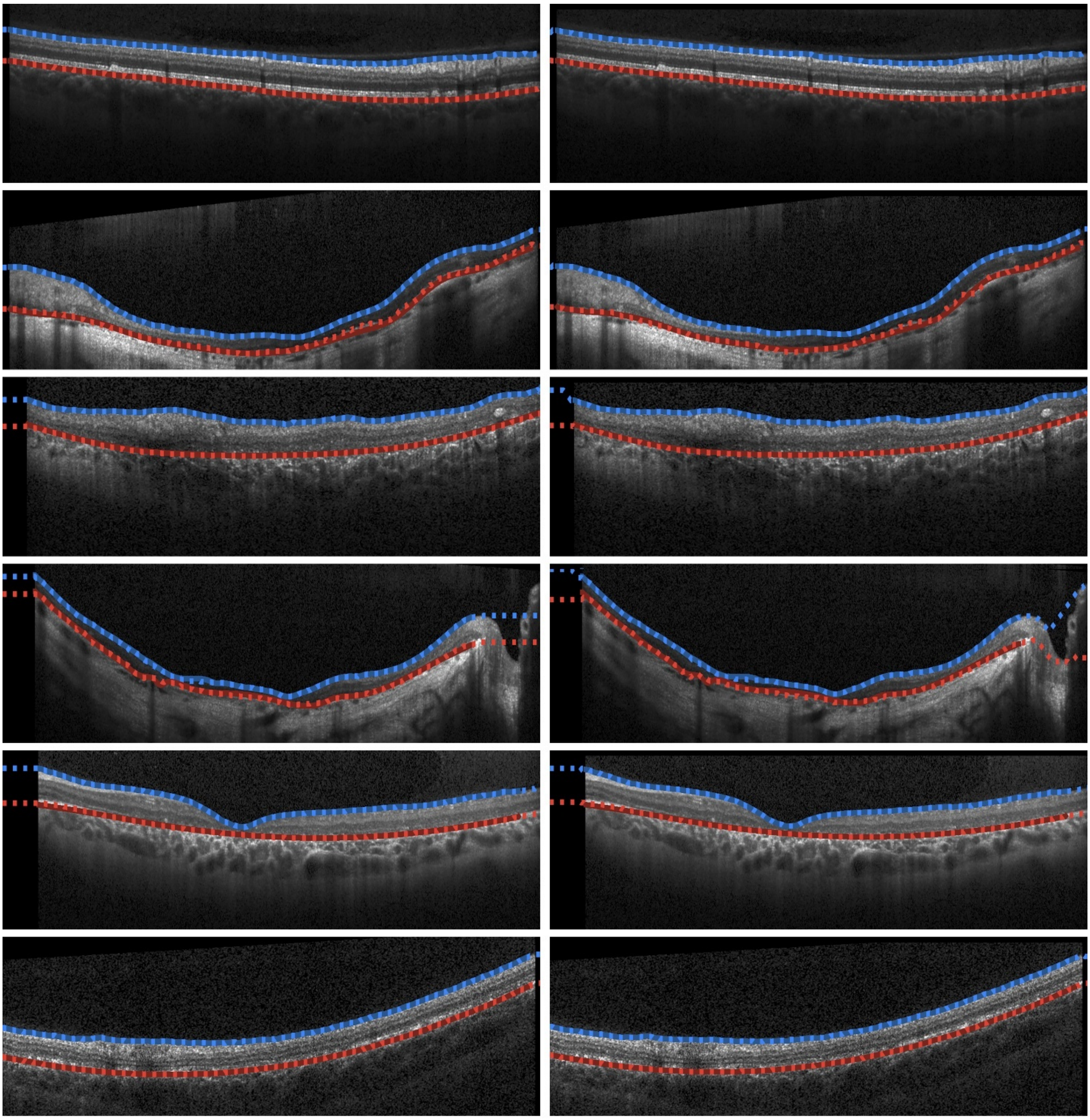

Supplementary Fig 3: Example segmentations of Stargardt OCT images from FS-GS (the proposed ML-based semantic segmentation method) (left) and those provided by the patch-based ML segmentation method (right). Blue: ILM, Red: RPE. Solid lines indicate the ground truth boundary locations while the dotted lines correspond to the predicted locations.
