# Supplementary material for: Retinal Boundary Segmentation in Stargardt Disease Optical Coherence Tomography Images Using Automated Deep Learning
Source: Transl Vis Sci Technol. 2020 Oct 13;9(11):12. doi: 10.1167/tvst.9.11.12 (PMC7581491; doi:10.1167/tvst.9.11.12)
Supplement: Supplement 4 [file tvst-9-11-12_s004.pdf]

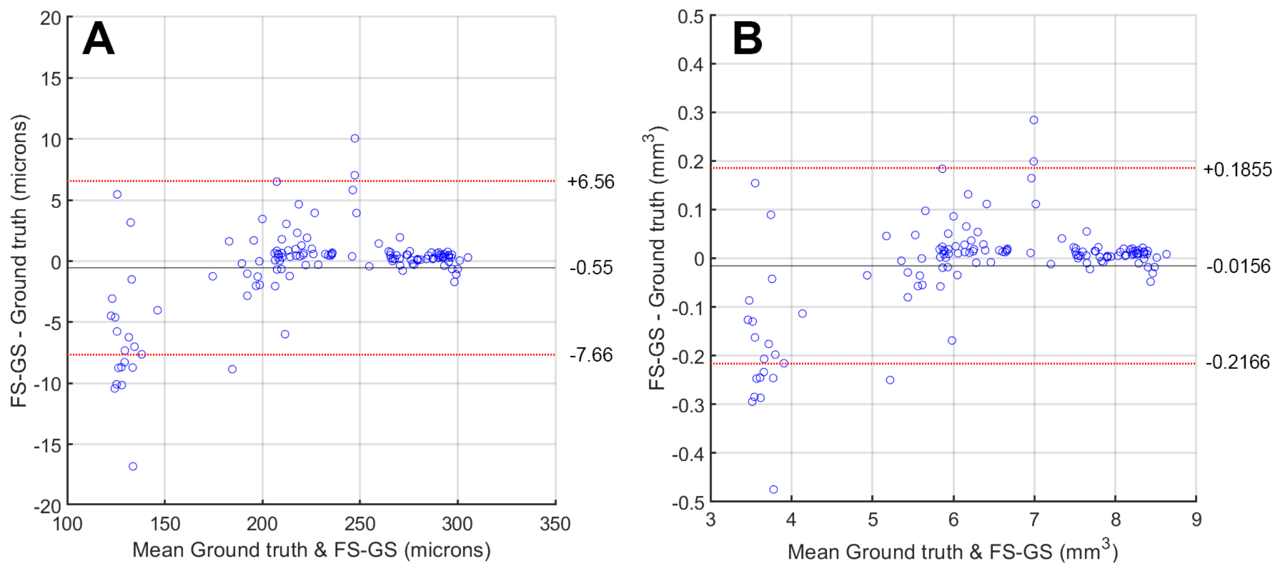

Supplementary Fig 4: Bland-Altman plots for retinal thickness (μm) [subplot A] and volume (mm^3) [subplot B] for FS-GS vs. ground truth across the whole 6mm diameter central zone. Limits of agreement are marked in red, mean difference in black with each individual volume (129 total) marked by a blue circle.
