# Supplementary material for: Retinal Boundary Segmentation in Stargardt Disease Optical Coherence Tomography Images Using Automated Deep Learning
Source: Transl Vis Sci Technol. 2020 Oct 13;9(11):12. doi: 10.1167/tvst.9.11.12 (PMC7581491; doi:10.1167/tvst.9.11.12)
Supplement: Supplement 5 [file tvst-9-11-12_s005.pdf]

Supplementary Table 1: Retina and overall Dice overlap (%) value for the average of the individual folds and the ensemble of all folds.

Method	Average		Ensemble	
	Retina Dice (SD) [%]	Overall Dice (SD) [%]	Retina Dice (SD) [%]	Overall Dice (SD) [%]
ON 4	96.71 (3.19)	97.49 (2.50)	96.70 (2.99)	97.50 (2.44)
OFF 4	96.72 (3.46)	97.54 (2.43)	96.72 (3.36)	97.54 (2.37)
ON 5	96.79 (3.01)	97.75 (2.28)	96.79 (2.86)	97.75 (2.20)
ON 4 scSE	96.75 (3.15)	97.66 (2.40)	96.76 (2.90)	97.65 (2.30)
ON 4 scSE NA	96.75 (2.82)	97.66 (2.36)	96.74 (2.97)	97.71 (2.25)

Each method was run five times with the average Dice and standard deviation across the five runs presented here. ON/OFF: whether contrast enhancement was used. 4/5: number of pooling layers used. scSE: squeeze + excitation blocks were incorporated. NA: no augmentations were utilised.
