# Supplementary material for: Retinal Boundary Segmentation in Stargardt Disease Optical Coherence Tomography Images Using Automated Deep Learning
Source: Transl Vis Sci Technol. 2020 Oct 13;9(11):12. doi: 10.1167/tvst.9.11.12 (PMC7581491; doi:10.1167/tvst.9.11.12)
Supplement: Supplement 6 [file tvst-9-11-12_s006.pdf]

The spatial squeeze and channel excitation (cSE) block variant operates on the feature maps in two stages: 1) spatial squeeze, and 2) channel recalibration/excitation. In the first stage, each input feature map is squeezed into a vector (i.e. to spatial dimensions of 1x1), using global average pooling. In the second step, this set of vectors is passed through a pair of dense layers followed by the sigmoid activation function to encode dependencies between channels. These activations are then used to recalibrate the original feature maps by weighting the relative importance of each channel. On the other hand, the channel squeeze and spatial excitation (sSE) block variant operates by recalibrating the feature maps to place greater emphasis on more relevant and important spatial locations. There are once again two stages: 1) channel squeeze, and 2) spatial recalibration/excitation. First, channel squeeze is performed using a convolutional layer with a 1x1 kernel and a single filter, reducing the number of channels to one while retaining the original spatial dimensions. Spatial recalibration is then achieved using the sigmoid activation function with each activation used for reweighting the individual spatial locations based on their relative importance. In our network, we consider the concurrent spatial and channel squeeze and excitation block variant (scSE) which combines the benefits of both the cSE and sSE blocks through element-wise addition of their outputs. More details for the function of the scSE block are illustrated in Supplementary Figure 1.
